# Initiating Insulin Pumps in Youth with New-onset Type 1 Diabetes: A Quality Improvement Initiative

**DOI:** 10.1097/pq9.0000000000000803

**Published:** 2025-03-19

**Authors:** Mili Vakharia, Sarah K Lyons, Don Buckingham, Mark Rittenhouse, Siripoom McKay, Rona Sonabend, Grace Kim

**Affiliations:** From the * Division of Pediatric Diabetes and Endocrinology, Department of Pediatrics, Baylor College of Medicine, Houston, Tex.; † Division of Pediatric Diabetes and Endocrinology, Nationwide Children’s Hospital, Columbus, Ohio.

## Abstract

**Introduction:**

Insulin pump therapy is recommended for youth with type 1 diabetes (T1D) as it enhances quality of life and improves glycemic management. We led a quality improvement initiative to increase insulin pump use in youth younger than 18 years of age with recently diagnosed T1D (duration <1 y) from a baseline of 17% to 27% from January 2021 to December 2023. As a balancing measure, we evaluated the diabetes-related ketoacidosis (DKA) rate in the same cohort as nonpump users.

**Methods:**

We implemented the following plan-do-study-act cycles: (1) development and implementation of pump initiation algorithm, including minimal safe start criteria and education on ketosis management with pump action plan, (2) establishing clinic follow-up within 90 days of pump start, (3) expansion of the pump algorithm at additional clinic locations, (4) early patient/caregiver education about pumps at a clinic visit 2 weeks after diagnosis, and (5) insulin pump therapy workshop for staff and providers.

**Results:**

There was a centerline shift in the percentage of patients with recently diagnosed T1D on insulin pumps from 17% to 28% from January 2021 to December 2023. We also found no pumps-related DKA encounters amongst patients with recently diagnosed T1D.

**Conclusions:**

Our improvement efforts increased pump usage in our cohort without related DKA events. A multidisciplinary approach with education on managing pumps should be implemented to prevent shortcomings such as DKA. Future directions are to evaluate HbA1c and pre-pump and post-pump DKA rates.

## INTRODUCTION

Insulin pump therapy is recommended for youth with type 1 diabetes (T1D) as it is associated with enhanced quality of life, improved glycemia, and decreased hypoglycemia.^1–5^ Unlike multiple daily injections, insulin pumps allow for alleviating diabetes burden by delivering precise, rapid-acting insulin throughout the day and enhancing glycemic control.^1,6^ There are some concerns for diabetes-related ketoacidosis (DKA), but the literature shows that the risk remains low in insulin pump users compared with those with multiple daily insulin injection users.^7–9^

Many institutions have implemented quality improvement (QI) efforts to increase pump use in pediatric patients. The T1D Exchange Quality Improvement Collaborative (T1DX-QI) collaborates among diabetes centers in the United States to enhance diabetes care delivery for patients with T1D.^9^ Through plan-do-study-act (PDSA) cycles led by T1DX-QI, the rate of insulin pump use in 12- to 26-year-olds increased from 45% to 58% for 22 months in 5 participating centers.^9^ Another single-center study decreased the time from decision to initiating pump starts from 132.5 to 98.5 days (*P* = 0.007) through various interventions, including optimizing patient education and support around technology.^10^

Other barriers to the integration of insulin pumps have also been identified, including lower socioeconomic status, lack of financial coverage, anxiety, depression, provider implicit bias, preexisting issues maintaining glycemic control, and lack of support.^11^ At our institution, we noted similar barriers to pump technology and, thus, worked toward improving access to pumps for our patients and families using a standardized process.

The literature review heavily guided the formulation and execution of our PDSA cycles. The American Diabetes Association and International Society Of Pediatric And Adolescent Diabetes 2022 recommend that adequate education to patients and multidisciplinary support are crucial to preventing adverse events, including DKA, pump malfunctions, infusion set failures, in patients using insulin pump therapy.^1,2,10,12,13^

At our institution, similar to the literature, we noted the underutilization of insulin pumps, and general prescribing barriers with a lack of standardized process for insulin pump starts. Our global aim was to standardize the insulin pump initiation process with safety, effectiveness, and patient-centered care goals. Our specific aim was to increase insulin pump use in youth younger than 18 years of age with recently diagnosed T1D (duration <1 y) from baseline of 17% to 27% from January 2021 to December 2023.

## METHODS

### Context

The setting is a free-standing, tertiary pediatric hospital with more than 900 inpatient beds, found in Houston, Texas. The Texas Children’s Hospital (TCH) Diabetes and Endocrine is one of the largest pediatric diabetes care centers in the United States, with access to four inpatient facilities and seven outpatient locations. About 360 newly diagnosed youth with T1D are admitted to TCH annually, contributing to more than 3,700 pediatric patients with T1D. Caring for pediatric patients with diabetes is a complex multidisciplinary process, with at least quarterly office visits with a physician or advanced practice provider and annual visits with diabetes educators, registered dietitians, and social workers.

### Interventions

We launched a QI project in January 2021. A robust multidisciplinary QI team, referred to as the Diabetes Education Care Process Team, consisting of members including endocrinologists, advance practice providers, diabetes educators, dietitians, and data analysts, met bi-weekly along with hospital leadership to drive this project. This group identified key barriers and challenges leading to pump starts in our cohort.

**Fig. 1.**
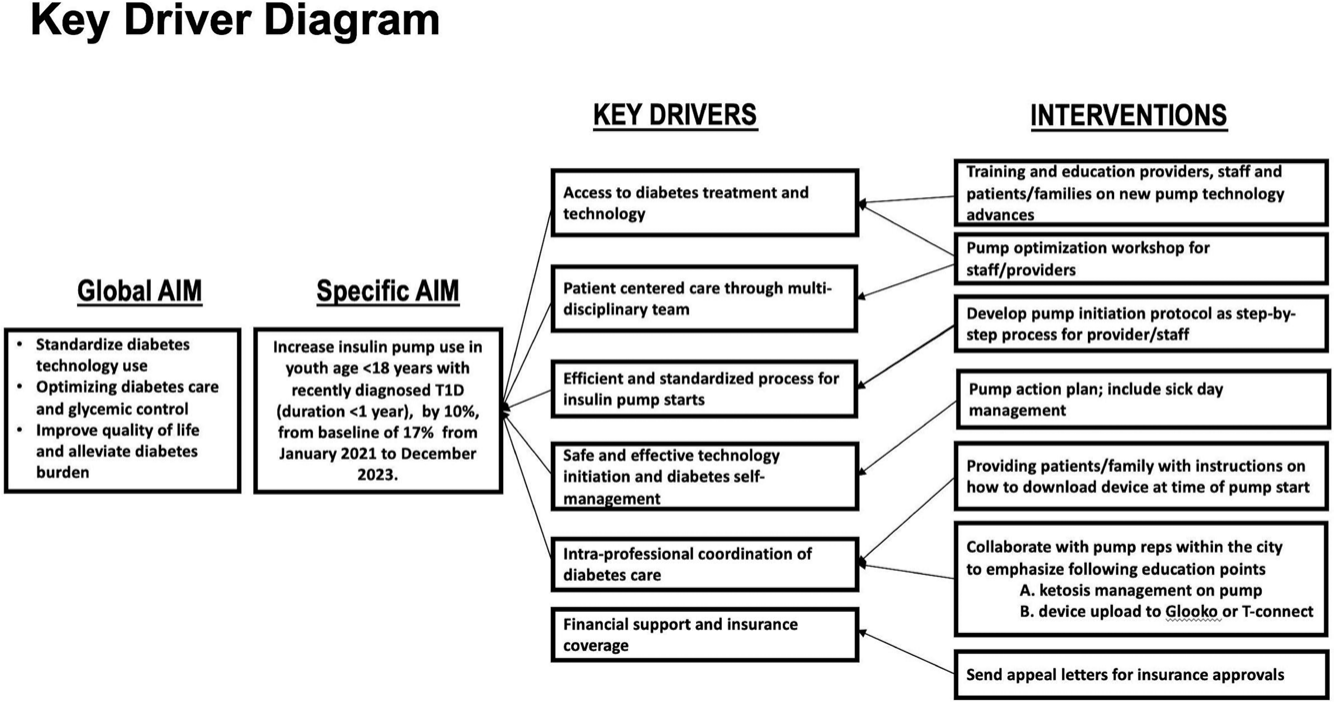
Key driver diagram showing interventions associated with the project.

Through stakeholder meetings and surveys, we learned about providers’ and staff members’ reasons for our institution’s lack of increased pump uptake. Some providers preferred certain HbA1c values and minimal risk of DKA, whereas others had an age criterion to start pump therapy. Other system-level barriers included a lack of departmental consensus regarding pump initiation and follow-up visits. As a balancing measure, we evaluated DKA rates in the same cohort as nonpump users. A key driver diagram helped identify factors contributing to project goals and interventions to attain those goals (Fig. 1).

**Fig. 2.**
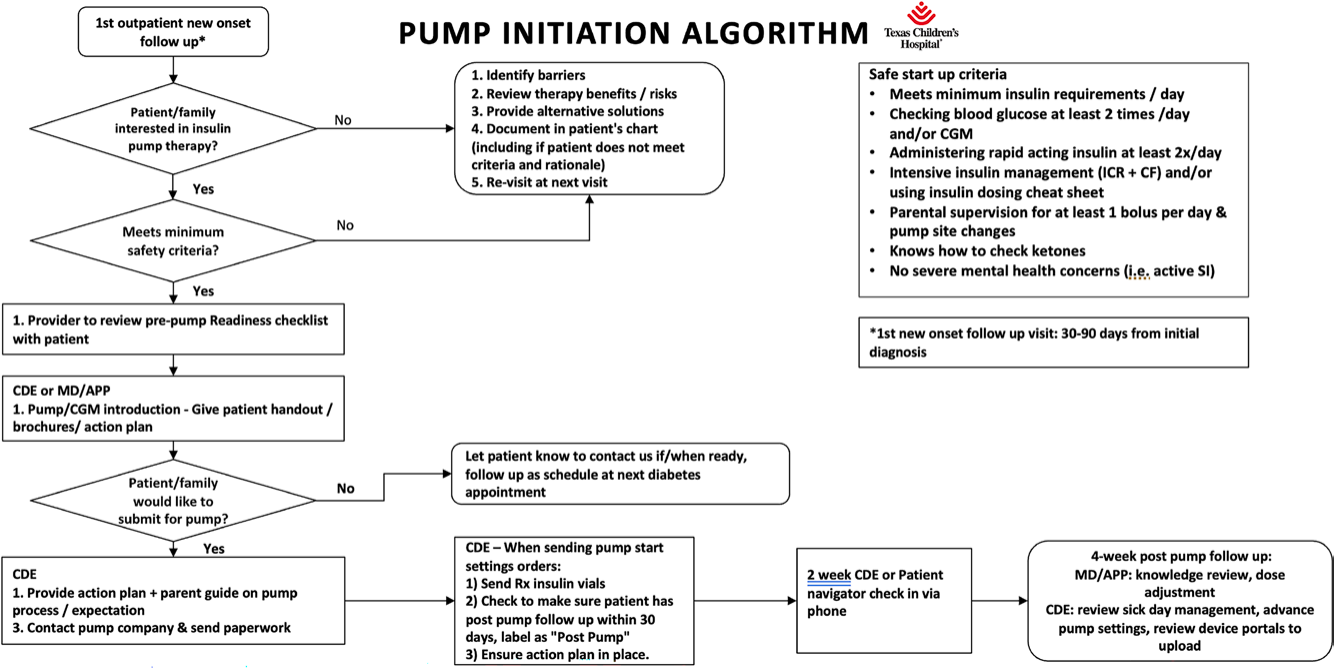
Pump start process map/algorithm.

**Fig. 3.**
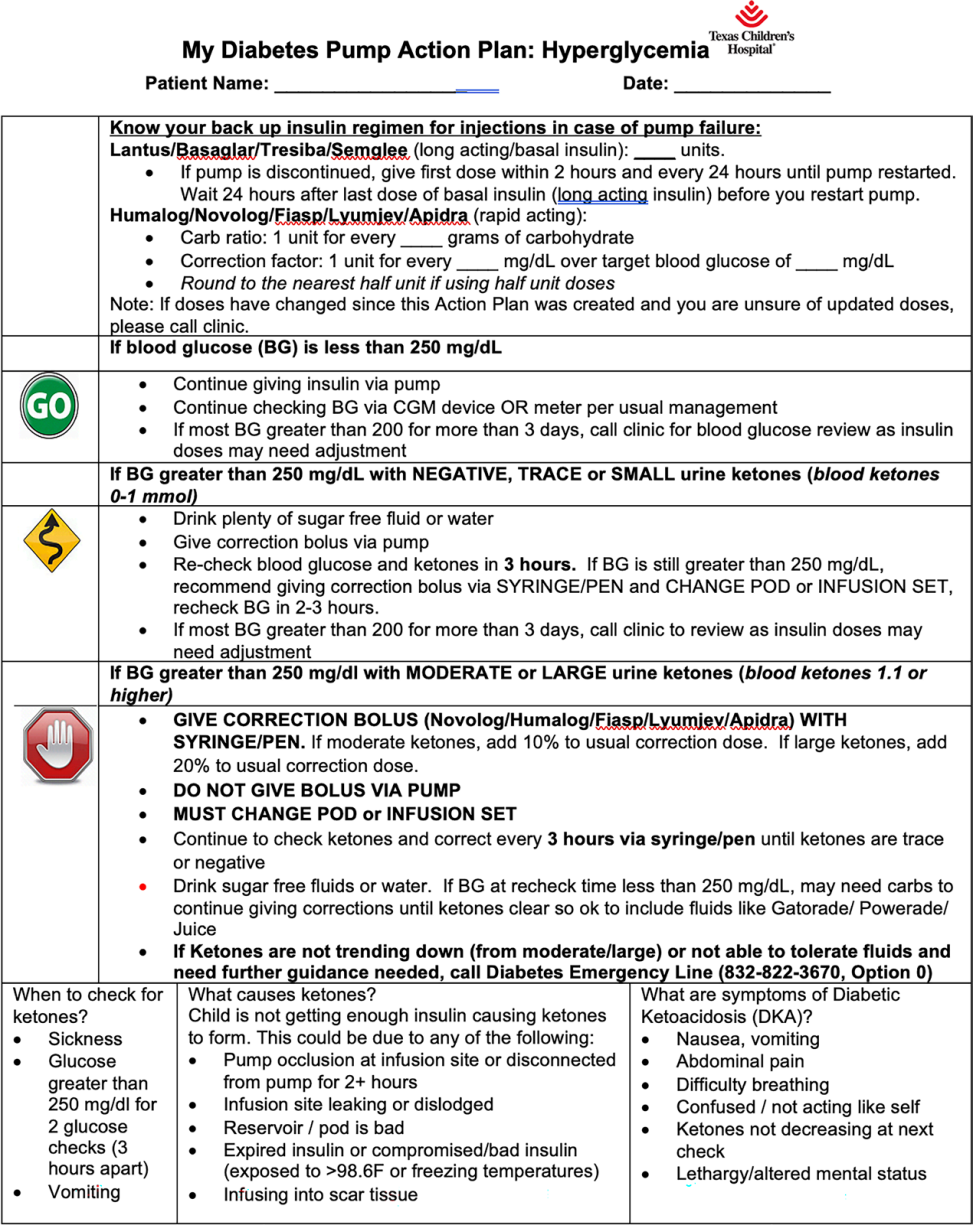
Pump action plan.

**Fig. 4.**
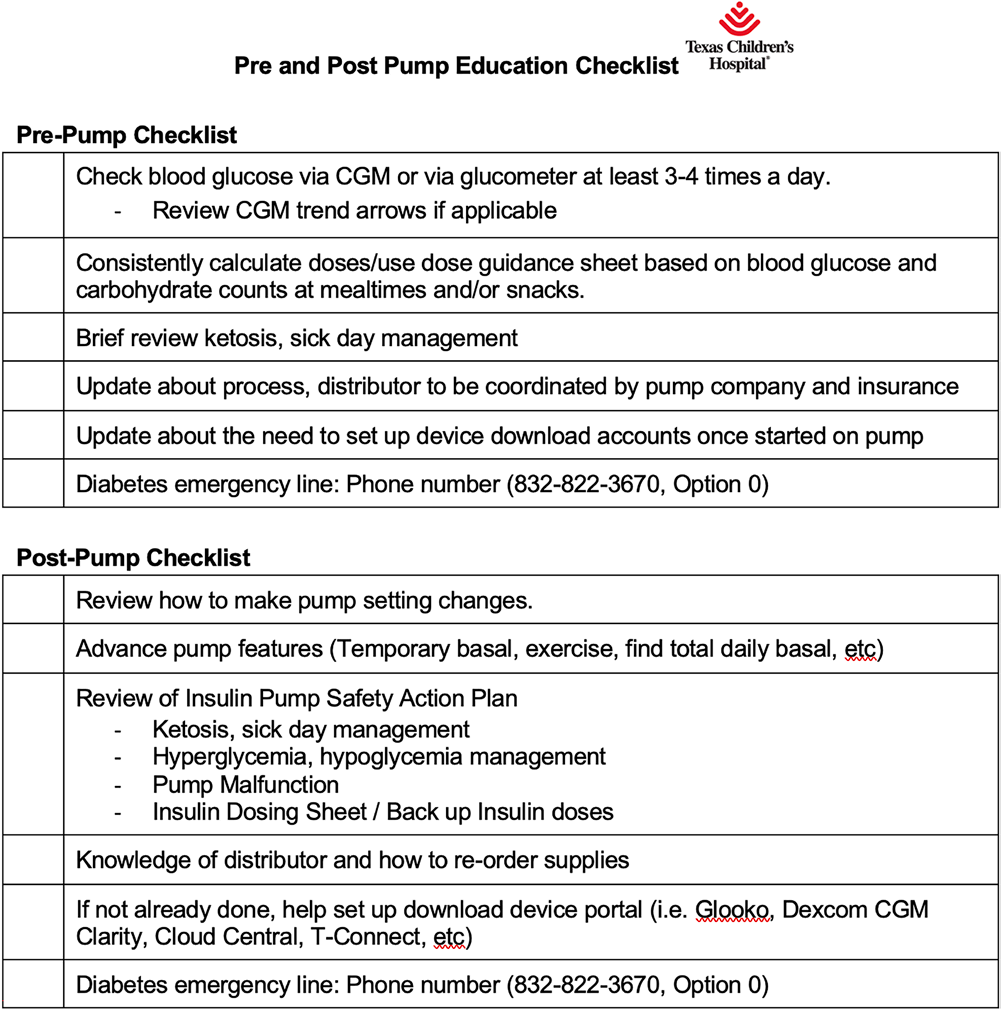
Prepump and postpump education checklist.

We implemented the following PDSA cycles:

1) Implementation of insulin pump initiation algorithm
In January 2021, the Diabetes Education Care Process Team members implemented an insulin pump initiation algorithm at 2 diabetes care clinics with minimal safe start criteria, pre-pump and post-pump education checklist, and associated education on ketosis management with a pump safety action plan (Figs. 2–4).
As such, all providers and staff across pilot clinics underwent training about the new pump start process. This process was later modified during the project from the initial start of insulin pump technology at 30 days to within 90 days of diabetes diagnosis. As the project progressed safely, we adapted the PDSA cycle to introduce pump start initiation as early as 2 weeks from diagnosis to 30 days after diagnosis. All our patients received pre-pump and post-pump education at their clinic visits.
2) Scheduling close post-pump follow-up visits
At each pump start, the team ensured patients had a scheduled follow-up clinic appointment and correctly labeled within 90 days of pump initiation. This appointment was conducted as a shared visit with the diabetes educator and provider to facilitate verification of adequate insulin pump settings, address knowledge gaps with sick day management, and troubleshoot device issues.
3) Spread the new standardized insulin pump start process to all campuses
In March 2022, we noted success with pump starts at our pilot clinic and, thus, expanded the pump algorithm to additional clinics within our system.
4) Utilizing telemedicine visits for earlier pump introduction
In September 2022, we launched introductory education on insulin pump technology to patients and caregivers at the 2-week post-diagnosis virtual clinic visit—an added change to the interventions to offer earlier access to diabetes technology for our patients and families.
5) Conducting an insulin pump workshop for providers and staff
Given evolving diabetes pump technologies, we conducted an insulin pump workshop in April 2023 for all clinical staff members and providers to revisit the pump start process and deliver education to help guide management.

### Study of Interventions

Baseline and subsequent data were extrapolated from our institution’s electronic medical record (EMR) (ie, Epic Systems, Verona, Wis.) and data analytics tool QlikView (Qlik, King of Prussia, Pa.). We placed a data request through our internal business analyst team, which was obtained from the provider flowsheet in Epic Systems. The data have been validated multiple times for accuracy purposes.

### Measures and Analysis

We used statistical process control charts to evaluate the primary outcomes and established rules to identify special cause variation. This project was undertaken as a QI initiative to improve diabetes care at TCH and is consistent with QI project guidelines at our institution. Using QI Macros Add-In for Excel software to generate a P Chart, we applied the Institute of Health Care statistical process control rules, stipulating that a consecutive series of 8 points above and below the centerline warrants a centerline shift.^14^

The primary outcome measure was the percentage of patients with newly diagnosed T1D placed on pump technology within 1 year. The process measures included a percentage of staff and providers trained about the new process of early pump technology adoption and implementation of the new pump process with smart phrases used. The pump initiation algorithm was designed after input from key stakeholders and helped reinforce the key educational points, such as ketone monitoring and pump troubleshooting.

The balancing measure was the number of admissions for DKA for patients initiated on insulin pump therapy. These data were obtained through EMR and manual chart reviews to verify accuracy. In addition, our project did not request extra support staff such as a designated diabetes educator or provider FTE (ie, full-time equivalents) to conduct the PDSA cycles and leverage existing resources and personnel in the setting of the post-pandemic era.

### Ethical Considerations

The institutional review board determined that this project was QI, not human subject research. Therefore, it did not require institutional board review and approval per institutional policy. We prepared this article using the Standards for Quality Improvement Reporting Excellence 2.0 guidelines.

## RESULTS

**Fig. 5.**
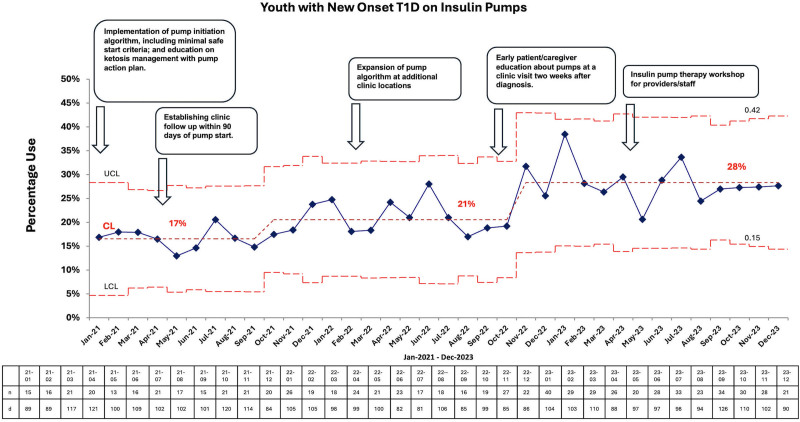
Outcome measure: percent increase in youth with new-onset T1D using insulin pumps. CL, centerline; LCL, lower control limit; UCL, upper control limit.

Between January 2021 and December 2023, the mean percentage of patients using insulin pumps within 1 year of diagnosis increased from 17% to 28% (Fig. 5). The statistical process control P Chart shows centerline shifts among patients with T1D who were seen for follow-up visits on insulin pump therapy within that month, with a diabetes duration of less than or equal to 1 year. The denominator was all other insulin modalities, including multiple daily dose injections.

Six months before implementing this project, the EMR data for our newly diagnosed youth with T1D utilizing insulin pump technology ranged from 11.1% to 17.6% between June 2020 and December 2020 (data not shown). The original mean centerline was 17% from January 2021 through September 2021, and then beginning in October 2021, a consecutive series of eight points above that centerline prompted a shift upward to a mean of 21%, and then again another shift in November 2022 to a new mean of 28%. Furthermore, applying the American Society of Quality’s rules (ie, run of 8 in a row on the same side of the centerline, or 10 of 11, 12 of 14, or 16 of 20) and the aggregate point rule,^15^ the subsequent data also confirmed these shifts to be appropriate. We did meet our project goal of increasing by an absolute 10% from baseline with a statistical change and improvement in the process, as noted in Figure 5.

Regarding the process measures, more than 80% of our staff and providers underwent training to operationalize the pump process. In addition, we had more than 100 attendees at the departmental pump workshop that reviewed the basics of different pump technologies available in the market, the benefits of pump technology, including lower risk of DKA, and information on pump troubleshooting. To assess pump safety, we also reviewed the number of DKA admissions for patients admitted to the hospital within 1 year of diagnosis. Per EMR data, 46 DKA encounters among patients with new-onset T1D followed in our clinic, and none were associated with pump concerns.

## DISCUSSION

Our QI efforts allowed for increased uptake of insulin pump therapy within 1 year of diabetes diagnosis without contributing to DKA events. A multidisciplinary approach with education on managing pumps could help prevent complications such as DKA.^13^ Early collaboration among our clinic staff, key stakeholders, and several team members was essential in driving QI efforts to improve the healthcare delivery for our patients. Insulin pump users also require education on managing acute issues such as pump site failure and hyperglycemia, and therefore, a pump safety action plan provided during our prepump and postpump visits likely helped mitigate pump-related diabetes emergencies.

After the initial PDSA cycle, we noted an increase in the uptake of pump technology, as shown in Figure 5. The pump initiation criteria were changed to a second follow-up visit, 90 days post-T1D diagnosis, due to a lack of discomfort from providers and staff members on earlier pump starts. To mitigate concerns about pump site or technology-related issues, we ensured that patients had a close post-pump follow-up with the provider and diabetes educator to assess for any knowledge gap, make dose adjustments, review sick day and ketosis management, review advance pump settings, and assist the patients with device portal uploads using the post-pump checklist shown in Figure 4.

The initial centerline shift occurred in October 2021 after scheduling a close follow-up visit for patients new to pump technology. We speculate that this intervention reassured our providers and staff members to safely implement the pump technology earlier in the patients' diabetes journey. In November 2022, we noted another centerline shift and improvement in our insulin pump usage particularly given our PDSA efforts with earlier introductions to the pump. Another remarkable uptake happened between May and June 2023, after the insulin pump workshop that reminded and re-educated providers and staff about the need to offer insulin pump technology prescriptions for all patients and families with minimal safe eligibility criteria.

Despite the known benefits of pump technology, the risk of DKA remains a concern when implementing pump technology.^1^ Multicenter studies have shown similar benefits with early adoption of pump technology in children diagnosed within 6 months with T1D, including lower HbA1c levels (*P* = 0.0006^16^; *P* < 0.0001^17^), reduced rates of hypoglycemia (*P* = 0.0064^16^), and milder or no DKA events.^16–18^

The American Diabetes Association and International Society of Pediatric and Adolescent Diabetes support structured education for patients using diabetes technology to optimize their success with device use.^1,2,12^ Focusing on standardizing the approach to early pump therapy initiation and multidisciplinary education led to increased sustainment of pump technology uptake in our cohort.^19^ This structured process could help mitigate unconscious prescriber biases contributing to healthcare disparities. Our preliminary post-pump workshop survey showed that providers were more inclined to prescribe insulin pump technologies at an earlier duration of diabetes diagnosis.

Although we successfully improved the use of insulin pump technology in our cohort of patients, the findings are not generalizable given it is a single-institution initiative. In addition, there are multiple other variables, such as the partial remission phase of diabetes that potentially contributed to the lack of DKA events given the reduced insulin requirements, the ability for patients to modulate their insulin and have enhanced beta cell preservation compared with those presenting with DKA.^20^

Future efforts will evaluate the pre-pump and post-pump DKA rates, hospitalizations, and HbA1c levels within the same subjects. Our project did not address the known disparities in insulin pump technology use for youth with T1D, such as limitations by insurance types, race, ethnicity, education level, and socioeconomic status, as noted in national registries.^21^ However, we plan to leverage the EMR to track these data, to better understand and address the impact of social drivers on technology uptake at our institution, and to compare the outcomes to national metrics.

## CONCLUSIONS

Our improvement efforts increased pump usage in our cohort without increasing DKA events. The partial remission phase of diabetes potentially contributed to preventing DKA. A multidisciplinary approach with structured education on managing pumps could help prevent complications such as DKA. The interventions that helped drive the increase in pump technology use included earlier pump introduction and close follow-up visits to ensure pump safety and address any technology-related issues.

## ACKNOWLEDGMENTS

The authors acknowledge the contributions of patients, families, and the diabetes care process team members, including the administration, who supported and assisted with the implementation of the pump initiative. They continue to strive to improve care and outcomes for people with diabetes.

## References

[1] Sherr JL Schoelwer M Dos Santos TJ. ISPAD clinical practice consensus guidelines 2022: diabetes technologies: insulin delivery. Pediatr Diabetes. 2022;23:1406–1431. 36468192 10.1111/pedi.13421

[2] American Diabetes Association Professional Practice Committee. 7. Diabetes technology: standards of care in diabetes-2024. Diabetes Care. 2024;47:S126–S144. 38078575 10.2337/dc24-S007 PMC10725813

[3] Aleppo G DeSalvo DJ Lauand F. Improvements in glycemic outcomes in 4738 children, adolescents, and adults with type 1 diabetes initiating a tubeless insulin management system. Diabetes Ther. 2023;14:593–610. 36763329 10.1007/s13300-023-01366-9 PMC9913031

[4] Karges B Schwandt A Heidtmann B. Association of insulin pump therapy vs insulin injection therapy with severe hypoglycemia, ketoacidosis, and glycemic control among children, adolescents, and young adults with type 1 diabetes. JAMA. 2017;318:1358–1366. 29049584 10.1001/jama.2017.13994 PMC5818842

[5] Foster NC Beck RW Miller KM; State of Type 1 Diabetes Management and Outcomes from the T1D Exchange in 2016-2018. Diabetes Technology Therapeutics. 2019;21:66–72. 30657336 10.1089/dia.2018.0384 PMC7061293

[6] Ramchandani N Ten S Anhalt H. Insulin pump therapy from the time of diagnosis of type 1 diabetes. Diabetes Technol Ther. 2006;8:663–670. 17109598 10.1089/dia.2006.8.663

[7] Doyle EA Weinzimer SA Tamborlane W. DKA prevention and insulin pumps: lessons learned from a large pediatric pump practice. Sci Diabetes Self Manag Care. 2022;48:476–482. 36129121 10.1177/26350106221125699

[8] Everett EM Copeland TP Moin T. Insulin pump-related inpatient admissions in a national sample of youth with type 1 diabetes. J Clin Endocrinol Metab. 2022;107:e2381–e2387. 35196382 10.1210/clinem/dgac047 PMC9113825

[9] Lyons SK Ebekozien O Garrity A; T1D Exchange Quality Improvement Collaborative Study Group. Increasing insulin pump use among 12- to 26-year-olds with type 1 diabetes: results from the T1D exchange quality improvement collaborative. Clin Diabetes. 2021;39:272–277. 34421202 10.2337/cd21-0027 PMC8329008

[10] Alonso GT Hink R De Georgeo MR. Improving the insulin pump initiation process for pediatric patients with type 1 diabetes through application of lean quality improvement methods. Perm J. 2018;22:17–147. 10.7812/TPP/17-147 PMC6045500 30005730

[11] Walker AF Hood KK Gurka MJ. Barriers to technology use and endocrinology care for underserved communities with type 1 diabetes. Diabetes Care. 2021;44:1480–1490. 34001535 10.2337/dc20-2753 PMC8323174

[12] Desrochers HR Schultz AT Laffel LM. Use of diabetes technology in children: role of structured education for young people with diabetes and families. Endocrinol Metab Clin North Am. 2020;49:19–35. 31980118 10.1016/j.ecl.2019.11.001 PMC7140592

[13] Heinemann L Fleming GA Petrie JR. Insulin pump risks and benefits: a clinical appraisal of pump safety standards, adverse event reporting and research needs. A joint statement of the European Association for the Study of Diabetes and the American Diabetes Association Diabetes Technology Working Group. Diabetologia. 2015;58:862–870. 25784563 10.1007/s00125-015-3513-z

[14] QI Macros. Not sure which control charts are used in healthcare? Available at https://www.qimacros.com/control-chart/healthcare-rules/. Accessed December 28, 2024.

[15] Wheeler TA Davis JT Brilli RJ. The aggregate point rule for identifying shifts on P charts and U charts. Pediatr Qual Saf. 2018;3:e103. 30584630 10.1097/pq9.0000000000000103 PMC6221583

[16] Kamrath C Tittel SR Kapellen TM. Early versus delayed insulin pump therapy in children with newly diagnosed type 1 diabetes: results from the multicenter, prospective diabetes follow-up DPV registry. Lancet Child Adolesc Health. 2021;5:17–25. 33253630 10.1016/S2352-4642(20)30339-4

[17] Lang EG King BR Miller MN. Initiation of insulin pump therapy in children at diagnosis of type 1 diabetes resulted in improved long-term glycemic control. Pediatr Diabetes. 2017;18:26–32. 26782779 10.1111/pedi.12357

[18] Wersäll JH Adolfsson P Forsander G. Insulin pump therapy is associated with higher rates of mild diabetic ketoacidosis compared to injection therapy: a 2-year Swedish national survey of children and adolescents with type 1 diabetes. Pediatr Diabetes. 2022;23:1038–1044. 35678764 10.1111/pedi.13377 PMC9796597

[19] Kirkendall ES Brady PW Corathers SD. Safer Type 1 diabetes care at home: SEIPS-based process mapping with parents and clinicians. Pediatr Qual Saf. 2023;8:e649. 38571735 10.1097/pq9.0000000000000649 PMC10990404

[20] Nwosu BU. Partial clinical remission of type 1 diabetes mellitus in children: clinical applications and challenges with its definitions. Eur Med J Diabetes. 2019;4:89–98. 31069088 PMC6502244

[21] Gandhi K Ebekozien O Noor N; T1D Exchange Quality Improvement Collaborative. Insulin pump utilization in 2017-2021 for more than 22,000 children and adults with type 1 diabetes: a multicenter observational study. Clin Diabetes. 2024;42:56–64. 38230341 10.2337/cd23-0055 PMC10788665

